# Design Methodology of Passive In-Line Relays for Molecular Communication in Flow-Induced Microfluidic Channel

**DOI:** 10.3390/bios11030065

**Published:** 2021-02-27

**Authors:** Puneet Manocha, Gitanjali Chandwani

**Affiliations:** 1 School of Medical Science & Technology, IIT Kharagpur, Kharagpur 721302, India; 2 Electronics and Communication Engineering Department, Thapar Institute of Engineering and Technology, Patiala 147001, India; gc.manocha@thapar.edu

**Keywords:** microfluid channel, molecular communication, relay

## Abstract

Molecular communication is a bioinspired communication that enables macro-scale, micro-scale and nano-scale devices to communicate with each other. The molecular communication system is prone to severe signal attenuation, dispersion and delay, which leads to performance degradation as the distance between two communicating devices increases. To mitigate these challenges, relays are used to establish reliable communication in microfluidic channels. Relay assisted molecular communication systems can also enable interconnection among various entities of the lab-on-chip for sharing information. Various relaying schemes have been proposed for reliable molecular communication systems, most of which lack practical feasibility. Thus, it is essential to design and develop relays that can be practically incorporated into the microfluidic channel. This paper presents a novel design of passive in-line relay for molecular communication system that can be easily embedded in the microfluidic channel and operate without external energy. Results show that geometric modification in the microfluidic channel can act as a relay and restore the degraded signal up-to 28%.

## 1. Introduction

Molecular Communication (MC) system uses molecules to encode, transmit and receive information between macro-scale, micro-scale and nano-scale devices. Although there has been immense advancement in theoretical research, there is a lack of practical studies in MC [1]. The major reason for the lack of working applications is because most analytical models describe inter-cell, intra-cell and in-vivo models of communication, and thus, their practical validation is not straightforward. This would require some in-vitro models under controlled conditions to validate the theoretical studies. Lab-on-Chip (LOC) offers the most potent platform for the implementation and validation of MC theories. Moreover, MC can enable interconnection among various entities of LOC for sharing information [2,3]. MC system is characterized by high propagation delay which is proportional to the square of the distance between transmitter (Tx) and receiver (Rx). Further, the concentration of information molecules that reach the Rx is inversely proportional to the cubic power of the distance between Tx and Rx. This induces performance degradation and unreliability in end-to-end communication. Another limitation in the performance of the MC system is channel attenuation, which limits the transmission range of nodes. To mitigate this challenge, an obvious technique adopted from wireless communication is the use of relays [4,5]. The importance of relay signaling can also be found in biological systems, such as in cell-to-cell communication, where information is relayed into the interior of the target cell [6], and the quorum sensing in bacteria [7]. Relay-assisted MC has been well explored from the information-theoretic perspective [8,9], which shows that introducing the relay in the MC system increases the capacity of the system. In order to mitigate the distance-decay effect of molecular signals, authors in [10] employ the Depleted Molecule Shift Keying (D-MoSK) to model a Decode-and-Forward (DF) relay communication scheme.

Authors in [11,12] show that the relay-assisted MC systems are capable of achieving a lower bit error rate (BER) than single-hop MC systems. The works in [13,14] focus on the optimum placement of relay(s) in the microfluidic channel to improve the error performance of the system. Authors in [15] proposed several relaying schemes to improve the transmission range of MC systems. In our previous work [16,17] we presented the analytical model of dielectrophoresis based relay system for aiding MC for LOC that can be fabricated using the MEMS technology and has been put to practical studies.

Most of the above studies model the relay nodes as a black box that is capable of amplifying or decoding and regenerating the received signal. It is generally assumed the relay nodes are already in-place between Tx and Rx and are calibrated for sensing the threshold concentration for reliable detection. Thus, most of the solutions proposed for employing relay in MC lack practical feasibility. Therefore, there is a need to look for a viable and practical solution for relaying in a microfluidic channel.

**Figure 1:**
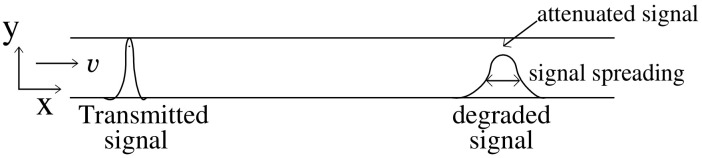
Concentration signal traversing microfluidic channel from Tx towards Rx degrades due to temporal spread and attenuation.

To the best of our knowledge, this is the first work that presents the novel idea of designing a passive in-line relay that can be easily incorporated into the microfluidic channel by modifying the geometry of the channel. As the concentration signal propagates in the microfluidic channel the peak concentration reduces with distance as shown in Figure 1. To maintain peak concentration above the detection threshold mark while propagating in the microfluidic channel, it is important to restore the concentration of the degraded signal. One of the possible solutions can be derived by approximating the degradation function for a microfluidic channel and designing the relay node which acts as a filter to recover the concentration signal. We propose to implement such a filter in the MC system by modifying the geometry of the microfluidic channel.

The major contributions of this paper are as follows: (1) we derive the degradation function which captures the effect of degradation due to dispersion and attenuation in the microfluidic channel. We analytically show that degradation suffered by a concentration signal in the microfluidic channel is equivalent to the impulse response of the turning microfluidic channel. (2) We model the Wiener filter to design the in-line passive relay for recovering the degraded signal, it is worth noting that this relay does not require any external energy source to restore the signal. (3) Finally, we simulate in-line passive relay, using FEM tool COMSOL Multiphysics 4.3a, to show that the degraded signal can be regained up-to 28% of the original concentration.

## 2. System Model

**Figure 2:**
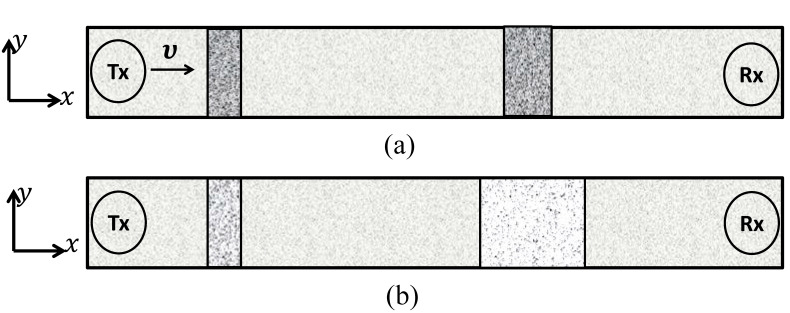
Propagation of the concentration signal from the transmitter (Tx) to the receiver (Rx) through the microfluidic channel based on the convection (**a**) and the diffusion (**b**).

The transport of molecules inside a microfluidic channel from the Tx to the Rx is illustrated in Figure 2a,b for the convection and the diffusion, respectively. Moreover, the convection can be steered through a confined microfluidic channel. The Hagen–Poiseuille flow is used for modeling flow in rectangular microfluidic channels, and it closely approximates a parabolic flow profile. However, the extent of similarity depends on the value of the aspect ratio (depth/width) of the lumen of the channel, the flow becomes flattened for channels with a higher aspect ratio(s) [18,19]. Thus, for ease of calculations, we consider a rectangular microfluidic channel with a higher aspect ratio and assume a flattened flow in the channel. Here we consider a long straight channel that is parallel to the *x* axis, the depth of the channel is parallel to the *y* axis. Consider a continuous flow of medium with velocity $\upsilon_{x}\left(t\right)$ along the *x* axis and $\upsilon_{y}\left(t\right)$ along the *y* axis due to which the concentration signal is subjected to planar motion. This motion causes the signal to degrade due to temporal spread and attenuation as it traverses in the microfluidic channel. The MC is set up in a two-dimensional microfluidic channel which is indexed by the two Cartesian axes x,y. We consider that the Tx is a point source placed at the origin (0,0) and emits $M_{o}$ number of information molecules that diffuse in the microfluidic channel for the time duration *T* seconds. Corresponding to the transmission by the Tx, the concentration c(x,y) of information molecules at time *t* at (x,y) coordinate is obtained as:

(1) $$c\left(x,y\right)=\frac{M_{o}}{2\pi \sigma^{2}}e^{-\left(\frac{x^{2}+y^{2}}{2\sigma^{2}}\right)}$$

where *D* is the diffusion coefficient and $\sigma =\sqrt{2Dt}$. In order to derive the degradation function, we assume that the concentration signal is subjected to time varying motion. Let $\left(\upsilon_{x}\left(t\right)\right)t=x_{o}\left(t\right)$ and $\left(\upsilon_{y}\left(t\right)\right)t=y_{o}\left(t\right)$ denote the time varying components of motion in *x* and *y* direction respectively. The degraded signal $c_{de}$ can be expressed as:

(2a) $$c_{de}\left(x,y\right)=\int_{t=0}^{T}c\left[x-x_{o}\left(t\right),y-y_{o}\left(t\right)\right]dt$$

(2b) $$c_{de}\left(x,y\right)=\int_{t=0}^{T}\frac{M_{o}}{2\pi \sigma^{2}}e^{-\left(\frac{{\left(x-x_{o}\left(t\right)\right)}^{2}+\left(y-y_{o}\left(t\right)\right){)}^{2}}{2\sigma^{2}}\right)}dt$$

The spatial Fourier transform of Equations (1) and (2b) respectively is expressed as:

(3a) $$C\left(\Omega_{x},\Omega_{y}\right)=\frac{M_{o}}{2\pi \sigma^{2}}e^{-\left(\Omega_{x}^{2}+\Omega_{y}^{2}\right)2\sigma^{2}}$$

(3b) $$C_{de}\left(\Omega_{x},\Omega_{y}\right)=\int_{-\infty }^{\infty }\int_{-\infty }^{\infty }c_{de}\left(x,y\right)e^{-j(\Omega_{x}x+\Omega_{y}y)}dxdy$$

where $\Omega_{x}$ and $\Omega_{y}$ are the spatial frequencies along the *x* and *y* axis, respectively. Substituting Equation (2a) into Equation (3b) and reversing the order of integration of Equation (4) is obtained, which is expressed as;

(4) $$\begin{array}{c} C_{de}\left(\Omega_{x},\Omega_{y}\right)=\int_{0}^{T}\left[\int_{-\infty }^{\infty }\int_{-\infty }^{\infty }c\left(x-x_{o}\left(t\right),y-y_{o}\left(t\right)\right)dxdy\right]e^{-j(\Omega_{x}x+\Omega_{y}y)}dt. \end{array}$$

with some mathematical modification of Equation (4), it can be expressed as:

(5) $$C_{de}\left(\Omega_{x},\Omega_{y}\right)=C\left(\Omega_{x},\Omega_{y}\right)\int_{t=0}^{T}e^{-j(\Omega_{x}x_{o}\left(t\right)+\Omega_{y}y_{o}\left(t\right))}dt$$

The above equation has two components: the Fourier transform of concentration signal and the function which degraded it. Thus, the degradation function can be expressed as:

(6) $$D\left(\Omega_{x},\Omega_{y}\right)=\int_{t=0}^{T}e^{-j(\Omega_{x}x_{o}\left(t\right)+\Omega_{y}y_{o}\left(t\right))}dt.$$

The peak concentration of the signal moves in both *x* and *y* directions. As stated earlier we assume a uniform flow, thus, the distance that the concentration signal covers is proportional to velocity. Therefore, the distance covered is $l=PeD/\upsilon_{x}\left(t\right)$ and $m=PeD/\upsilon_{y}\left(t\right)$, (Pe is Peclet number) in the *x* and *y* direction respectively. Further, substituting ϕ in the above expression, where $\phi =(l\Omega_{x}+m\Omega_{y})$, the degradation function can be solved as:

(7) $$D\left(\Omega_{x},\Omega_{y}\right)=\frac{2T}{\phi }\sin\left[\frac{\phi }{2}\right]e^{-j\frac{\phi }{2}}=T\mathrm{sinc}\left(\frac{\phi }{2}\right)e^{-j(\frac{\phi }{2})}.$$

Moreover, the attenuation inside the microfluidic channel is frequency-dependent. To account for this frequency-based attenuation, we multiply Equation (7) by $e^{-\beta \phi }$ with β being the static attenuation factor:

(8) $$D\left(\Omega_{x},\Omega_{y}\right)=T\mathrm{sinc}\left(\phi /2\right)e^{-\phi (\beta +j/2)}.$$

**Figure 3:**
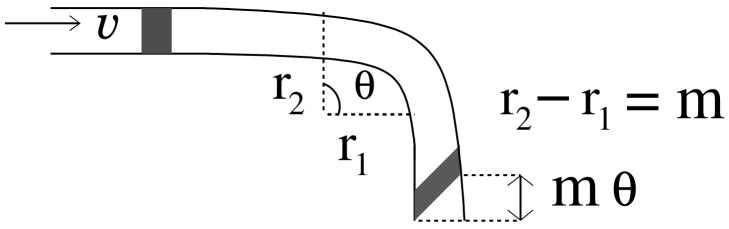
Signal propagation in a microfluidic channel with a turn.

Thus, Equation (5) can finally be expressed as,

(9) $$\begin{array}{cc} C_{de}\left(\Omega_{x},\Omega_{y}\right) & =C\left(\Omega_{x},\Omega_{y}\right)D\left(\Omega_{x},\Omega_{y}\right)\\ C_{de}\left(\Omega_{x},\Omega_{y}\right) & =C\left(\Omega_{x},\Omega_{y}\right)T\mathrm{sinc}\left(\phi /2\right)e^{-\phi (\beta +j/2)}\\ \; & \;\;\;\;\;\;\;\;\;+N(\Omega_{x},\Omega_{y}) \end{array}$$

where $N(\Omega_{x},\Omega_{y})$ is additive noise present in the microfluidic channel. Authors in [20] show that the impulse response of a turning channel is of the form $e^{-(D\Omega_{x}^{2}+j\Omega_{x}\upsilon )\tau }\mathrm{sinc}\left(\frac{m\theta \Omega_{x}}{2}\right)$, where exponential term is the impulse response of a straight channel and $\mathrm{sinc}(\frac{m\theta \Omega_{x}}{2})$ is function of angle and change in length due to the turn as shown in Figure 3. Comparing it with Equation (8), we can state that the degradation of the signal traversing in the microfluidic channel due to planar motion and frequency-based attenuation is similar to the impulse response of a turning microfluidic channel, where the turn is defined by an angle ϕ/2.

## 3. Design of Passive In-Line Relay

The relay receives a degraded signal, and to estimate the original signal $C(\Omega_{x},\Omega_{y})$ the estimator $\hat{C}\left(\Omega_{x},\Omega_{y}\right)$ should have minimum mean square error $\left(E_{r}^{2}\right)$

(10) $$\min\left(E_{r}^{2}\right)=\int_{-\infty }^{\infty }\int_{-\infty }^{\infty }{\left|C\left(\Omega_{x},\Omega_{y}\right)-\hat{C}\left(\Omega_{x},\Omega_{y}\right)\right|}^{2}.$$

For this purpose, we consider Wiener filter with transfer function expressed in Equation (12). The output of the filter is expressed as:

(11) $$\hat{C}\left(\Omega_{x},\Omega_{y}\right)=C_{de}\left(\Omega_{x},\Omega_{y}\right)H\left(\Omega_{x},\Omega_{y}\right),$$

where $H(\Omega_{x},\Omega_{y})$ can be expressed as;

(12) $$H\left(\Omega_{x},\Omega_{y}\right)=\frac{1}{D(\Omega_{x},\Omega_{y})}\left(\frac{|D\left(\Omega_{x},\Omega_{y}\right){|}^{2}}{|D\left(\Omega_{x},\Omega_{y}\right){|}^{2}+\frac{|N\left(\Omega_{x},\Omega_{y}\right){|}^{2}}{|C\left(\Omega_{x},\Omega_{y}\right){|}^{2}}}\right).$$

**Figure 4:**
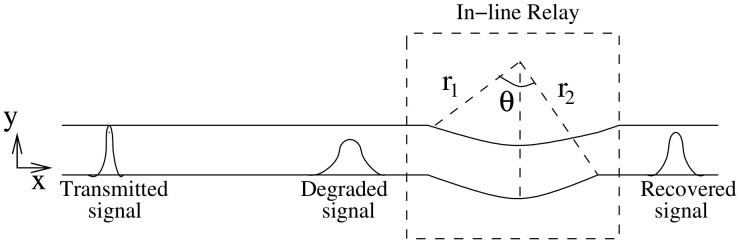
Concentration signal degrades as it traverses in straight microfluidic channel; inward turn of angle θ in the microfluidic channel restores the signal.

For reliable communication, the square of noise to signal ratio (i.e., 2nd term in the denominator) should tend to zero. Further, using Equation (8), we can expressed Equation (12) as:

(13) $$H\left(\Omega_{x},\Omega_{y}\right)\approx (D\left(\Omega_{x},\Omega_{y}\right){)}^{-1}=\frac{e^{\phi (\beta +j/2)}}{T\mathrm{sinc}(\phi /2)}.$$

where ${\left(D\left(\Omega_{x},\Omega_{y}\right)\right)}^{-1}$ is inverse of the degradation function. Since the derived degradation function is analogous to the impulse response of a turning channel, thus, the inverse of degradation function suggest that a complementary turn (i.e., a turn in inward direction) if placed in the line of a straight channel can restore the degraded signal to some extent. Therefore, we propose to use this complementary turn as an in-line passive relay. The relay is passive as it does not require any external energy source to restore the signal. It can be incorporated in the microfluidic channel by modifying its geometry i.e., introducing an inward turn in the microfluidic channel at the coordinates where the relay is to be placed, as shown in Figure 4. The turn is defined by the following parameters: (i) Angle θ subtended between the perpendicular from the inner curve and the outer curve of the microfluidic channel as shown in Figure 4, and (ii) Differential radius *r* which is defined as $r_{2}-r_{1}$. It is assumed that the channel width *m* is small compared to length *l* ( m≪l) and the channel is a constant width channel i.e., r=m. Thus, comparing Equation (8) with impulse response of turning channel we get:

(14) $$\phi \approx \Omega_{x}l=\frac{m\theta \Omega_{x}}{2\pi }.$$

(15) $$\theta =\frac{\pi l}{m}$$

where *m* is the width of the microfluidic channel, *l* is the length traversed by the signal in the microfluidic channel. It can be seen in the above equations, the angle of the turn is proportional to the distance traveled by the signal in the *x* direction and inversely proportional to the width of the channel. For complementary turn the angle $\theta_{c}=\pi -\theta$.

## 4. Results and Discussion

**Figure 5:**
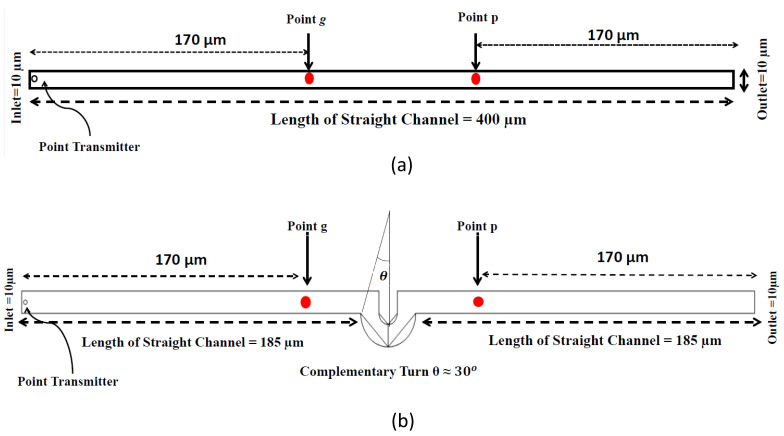
Geometry of (**a**) Straight Channel, (**b**) Inward turn (created using FEM software COMSOL Multiphysics 4.3a).

**Figure 6:**
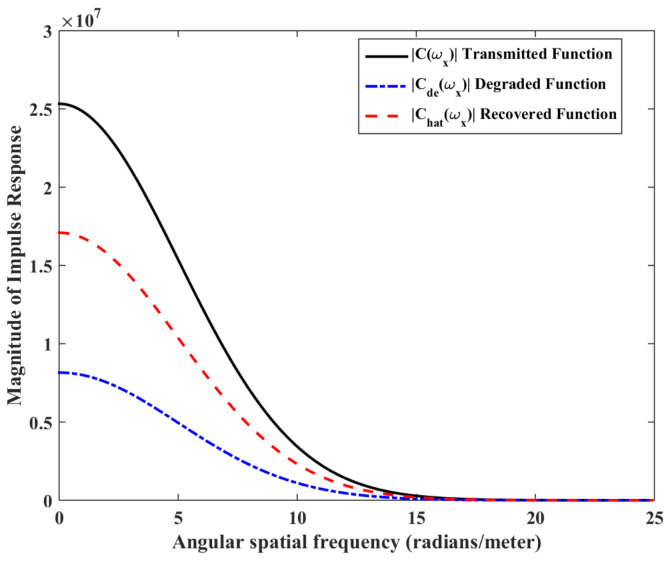
Impulse response magnitude versus spatial frequency of transmitted signal, degraded signal and recovered signal.

**Figure 7:**
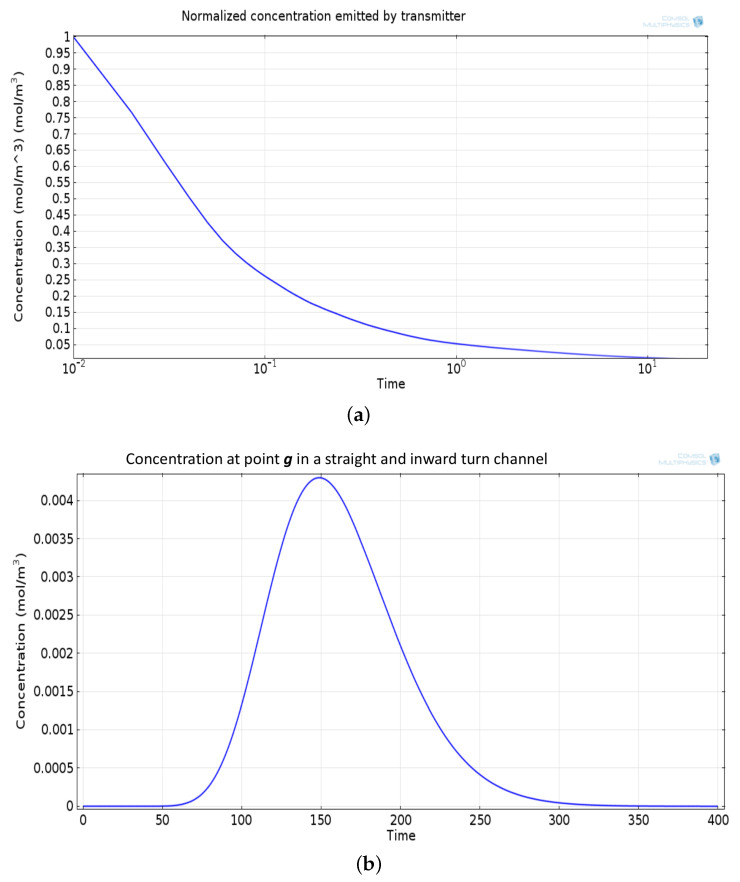
(**a**) Normalized concentration emitted by Tx. (**b**) Concentration signal at the point *g*, temporally spread and attenuated signal.

**Figure 8:**
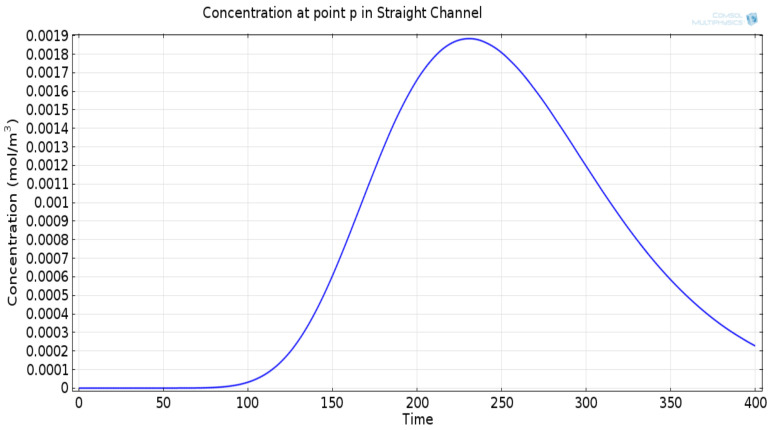

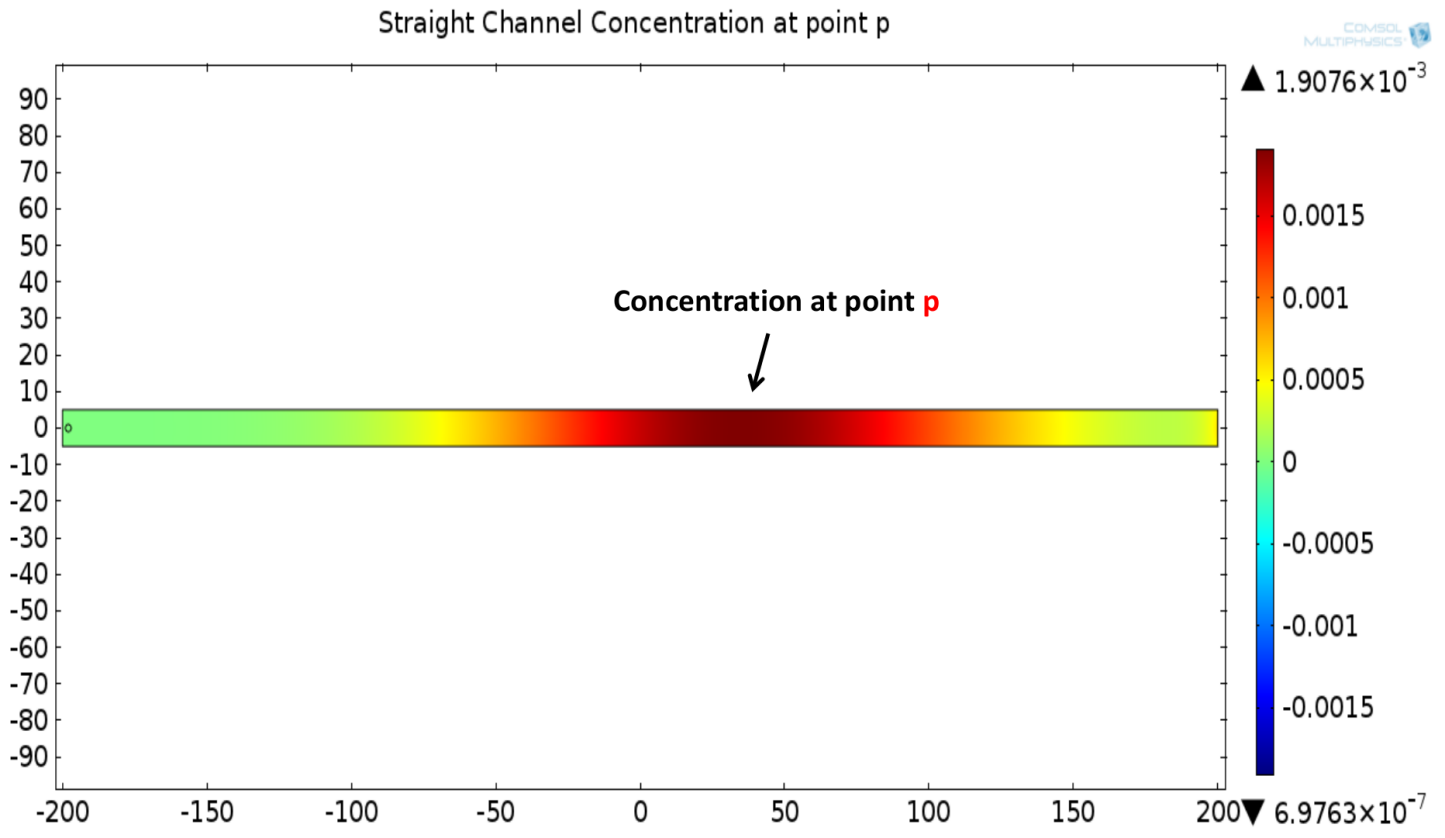
(**a**) Degraded concentration signal at the point *p* in a straight channel, temporally spread and attenuated signal. (**b**) Two-dimensional surface plot showing concentration profile at point *p* in a straight channel.

**Figure 9:**
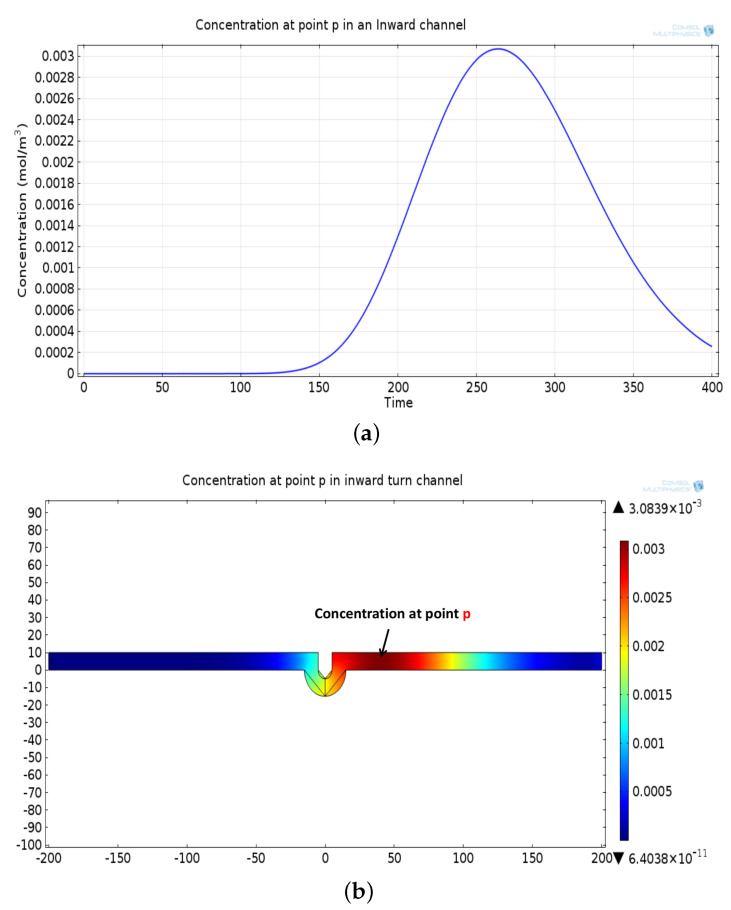
(**a**) Recovered concentration signal at point *p*, after passing through the outlet of inward turn. (**b**) Two-dimensional surface plot showing concentration profile at point *p* in a inward turn channel.

The mathematical model used for deriving the degradation function and the simulation model to show its effect in a channel is set up in 2D environment. In order to simulate the effect of a small inward turn placed in the midway of a straight channel, we use commercially available FEM tool COMSOL Multiphysics 4.3a. We use two geometries of the microfluidic channel: (i) straight channel and (ii) straight channel with an inward turn in middle shown in Figure 5a,b respectively. The parameters considered for the simulation are as follows: The length of the straight channel is 400 μm and width 10 μm with a point Tx placed at the inlet of both the channels (Tx is considered as point source because the diameter of Tx ≪ width of the channel). The inward turn geometry has a straight channel of length 370 μm (excluding the inward turn length), the width of 10 μm. An inward turn of angle 30 degrees is placed at 185 μm away from the inlet of the straight channel. In order to compare the inward turn channel and straight channel, we have considered two points *m* and *p*. These points are identically placed in both the geometries. The coordinates of these points have been chosen in such a way that, the point *g* is located near the inlet of inward turn, and the point *p* is placed near the outlet of the inward turn as shown in Figure 5b. The theoretical result presented in Figure 6 shows the magnitude of the impulse response of the transmitted signal in black solid-line, and the degraded signal after traversing distance *l* = 170 μm in blue dash-dot-line. It can be seen that the peak concentration of degraded signal drops to 35% of transmitted peak concentration. The red dashed-line in Figure 6 shows the signal recovered by Wiener filter $H(\Omega_{x},\Omega_{y})$, where the square of noise to signal ratio is set to 0.05. It is observed that the signal regains up to 66% of the transmitted peak concentration after introducing the in-line passive relay. Next, we present the simulated results obtained using the FEM tool COMSOL Multiphysics 4.3a. The physics of transport of diluted species and laminar flow in shallow channel approximation are jointly used, which are coupled through the velocity field. The transport of diluted species solves time-dependent convection and diffusion equation. The simulation was performed for t=400 s at an interval of Δt=1 s. The initial concentration at t=0 all boundaries (i.e., inlet, outlet, boundary walls) except that of point Tx placed at the inlet is considered to be zero. The initial concentration of point Tx at the inlet at t=0 is considered to be 1 mol/m${}^{3}$. The diffusion constant D is set to $10^{-11}$ m${}^{2}$/s as it is comparable to many biological molecules. All other boundaries are considered as a no-flux wall. The laminar flow physics solves time-dependent Navier–Stokes equations for the shallow channel. The medium considered is aqueous and the normal flow velocity is set at 1 μm/s. All initial conditions are considered to be zero and all boundaries are considered to be a no-slip wall. The physics-controlled mesh generation option in COMSOL was used to create the finite element grid, with a mesh size of normal. Other parameters used in the simulation are already mentioned at the beginning of this section. Figure 7a shows the concentration emitted by a point source Tx placed at the inlet of the geometry shown in Figure 5b and Figure 7b shows the concentration at point *g* for both the channels which is 0.0043 mol/m${}^{3}$. It can be observed that the concentration signal has degraded temporally and attenuated in strength with respect to the concentration signal emitted by Tx at the inlet. Figure 8a shows the concentration at point *p* of the straight channel, it shows that the concentration signal has further degraded and reduced to 41% of the signal strength present at point *g*. Figure 8b reflects the same interpretation in the 2D surface plot. The results in Figure 9a,b shows that the concentration value at point *p* (i.e., when the flow has exited the inward turn through an outlet and reached at a point *p*). It is observed that signal strength is 69% of the signal strength present at point *g*. Thus, it can be assumed that the degraded signal is restored after passing through the inward turn and the simulated results are found to be consistent with the theoretical results.

## 5. Conclusions

In this work, the degradation in the signal traversing in the straight microfluidic channel is approximated to the impulse response of the signal in a turning microfluidic channel. The concentration of transmitted molecules degrades while traversing in a microfluidic channel. The analytical and simulated result presented in this work shows that introducing a complementary turn in the microfluidic channel helps in restoring the degraded concentration. Hence, the complementary turn can act as a relay that works without any external energy to restore the signal. However, this increases the delay in the channel as incorporating turns in the microfluidic channel also increases the length of the channel. Thus, the number of such relays to be incorporated in the microfluidic channel should be optimized. Further, the turn geometry can also be optimized to achieve higher gain.

## Data Availability

Data sharing is not applicable to this article.
